# Questionnaire Survey of Possible Association of Allergic Diseases with Adverse Reactions to SARS-CoV-2 Vaccination

**DOI:** 10.3390/vaccines9121421

**Published:** 2021-12-01

**Authors:** Hiroki Morimoto, Satoshi Hayano, Naoya Ozawa, Yasuaki Ogura, Hiroaki Usui, Takahiro Usami, Ayako Ohse, Masaki Otsuka, Masahiko Miyachi, Yoshiki Tokura

**Affiliations:** 1 Department of Dermatology & Skin Oncology, Chutoen General Medical Center, Kakegawa 436-0040, Japan; y.ogura523@gmail.com (Y.O.); masaki-o@chutoen-hp.shizuoka.jp (M.O.); 2 Allergic Disease Research Center, Chutoen General Medical Center, Kakegawa 436-0040, Japan; jacafeva@gmail.com (S.H.); naoya0909@med.nagoya-u.ac.jp (N.O.); h-usui777fever@yc4.so-net.ne.jp (H.U.); usataka3808@yahoo.co.jp (T.U.); BXU03326@nifty.com (A.O.); 3 Department of Pediatrics, Chutoen General Medical Center, Kakegawa 436-0040, Japan; 4 Department of Respiratory Internal Medicine, Chutoen General Medical Center, Kakegawa 436-0040, Japan; 5 Department of Otolaryngology, Chutoen General Medical Center, Kakegawa 436-0040, Japan; 6 Department of Ophthalmology, Chutoen General Medical Center, Kakegawa 436-0040, Japan; 7 Department of General Practice, Chutoen General Medical Center, Kakegawa 436-0040, Japan; 8 Chutoen General Medical Center, Kakegawa 436-0040, Japan; mmiyachi@chutoen-hp.shizuoka.jp

**Keywords:** adverse effect, asthma, atopic dermatitis, food allergy, SARS-CoV-2 vaccine

## Abstract

To protect against COVID-19, SARS-CoV-2 vaccines have been widely used. Besides anaphylaxis, some less severe adverse effects may occur at higher frequencies. It remains unclear whether present or past histories of allergic diseases exert effects on local and systemic reactions. We conducted a questionnaire survey among workers in our hospital. We analyzed the adverse effects occurring after the first and second doses of the Pfizer–BioNTech vaccine in 955 subjects. The presence or absence of local injection reactions and systemic reactions (headache, fatigability, fever, muscle pain, and joint pain) was questioned. The intensities of these reactions were graded on a scale of 0–4 (except fever) or 0–2 (fever). The allergic diseases that we focused on were bronchial asthma, atopic dermatitis, food allergy, pollinosis, and hand eczema. For the systemic reactions, fatigability after the first dose tended to be more severe in the bronchial asthma than in the non-allergic group. Headache, joint pain, and fever tended to be more severe in the food allergy than in the non-allergic group after the second dose. For the local skin reactions, atopic dermatitis subjects tended to show rather less severe local skin reactions after the second dose. The results contribute to the guidelines for the care of individuals with different allergy histories, so that they may safely receive their vaccine.

## 1. Introduction

Severe acute respiratory syndrome coronavirus 2 (SARS-CoV-2) causes the highly infectious disease referred to as COVID-19, which gives rise to severe respiratory illness and other various manifestations [1]. An efficacious vaccine is essential to prevent further morbidity and mortality. Recently, several vaccines against SARS-CoV-2 have begun to be used worldwide, and it is evident that they can reduce the infection rate, disease severity, and transmission rate and contribute to disease control [2,3]. 

A vaccine from Pfizer–BioNTech has been approved in many countries and has been used as a leading reagent in Japan. The FDA EUA guidance for both the Pfizer–BioNTech and the Moderna vaccines is to prevent the administration of the vaccine to individuals with a known history of severe allergic reactions, such as anaphylaxis [4]. Careful prevention of anaphylaxis is recommended [5]. 

Besides anaphylaxis, some less severe adverse effects may occur at higher frequencies in the vaccine recipients, including pain, erythema, swelling, and pruritus in the vaccinated arm; and fever/chills, fatigability, muscle pain, and headaches as systemic symptoms [6]. These adverse reactions are different from rare allergic reactions, such as anaphylaxis, and have been reported to be more marked after the second dose [3].

Reports of possible anaphylactic or other adverse reactions have raised public concern. When people have experienced episodes of anaphylaxis, they should be careful when considering vaccination. However, it remains incompletely elucidated whether the adverse reactions are affected by other allergic conditions, including bronchial asthma (BA), atopic dermatitis (AD), food allergy (FA), and allergic rhinitis as represented by pollinosis. When vaccinating individuals, the doctor’s consultation of the medical interview sheet may sometimes be a time-consuming step. Although there is no great concern regarding these allergic diseases in relation to vaccination, there has been no definite study focusing on this issue. Allergists must offer clear guidance to individuals based on the best information available. 

In this study, we conducted a questionnaire survey among 1084 workers in our hospital, and 959 responses were recovered and analyzed. We investigated the adverse effects following the first and second doses of the Pfizer–BioNTech vaccine. The presence (and grade) or absence of local injection reactions and systemic effects was questioned, along with the present and past histories of allergic diseases. This study can be used for vaccine allergy epidemiology and as the basis of expert opinions, in conjunction with the guidance of public health and regulatory authorities.

## 2. Materials and Methods

### 2.1. Design

An observational questionnaire survey was performed on 8–15 June 2021. The questionnaire sheets were sent to 1084 workers in the Chutoen General Medical Center, Kakegawa, Japan, including physicians, nurses, and other healthcare workers. They were vaccinated with the Cominaty^®^ (Pfizer–BioNTech) vaccine in two doses at an interval of 21 days in our hospital. We obtained 955 responses (recovery rate, 88.1%). Our questionnaire survey was performed anonymously by filling in the sheet in Japanese (Supplementary Table S1; shown as the English version). We investigated the adverse effects following the first and second doses of the vaccine. The presence or absence of local injection reactions (pain, erythema, swelling, and pruritus) and systemic reactions (headache, fatigability, fever, muscle pain, and joint pain) was questioned. The intensities of these reactions were graded as none (0), mild (1), moderate (2), high (3), and severe (4). Fever was graded as <37.4 °C (0), 37.5°C–37.9°C (1), and 38.0 °C < (2). We also asked whether questionees had experienced anaphylaxis, though it is highly likely this item covered not only anaphylaxis but also vagal reflexes and other reactions.

The present and past histories of allergic diseases, including BA, AD, FA, pollinosis, and hand eczema, were checked. Hand eczema was included because it may represent a mild form of AD. Other allergic diseases, if any, were also noted. We evaluated differences in the local and systemic adverse effects between recipients who had present or past histories of allergic diseases and those without allergic diseases.

### 2.2. Primary Endpoint

The aim of the study was to evaluate the differences in vaccine-evoked, local injection reactions (pain, erythema, swelling, and pruritus) and systemic reactions (headache, fatigability, fever, muscle pain, and joint pain) between recipients who had present and past histories of allergic diseases (BA, AD, FA, pollinosis, and hand eczema) and those without any of these allergic diseases.

### 2.3. Population

Inclusion criteria were employment at the Chutoen General Medical Hospital, receipt of the Cominaty^®^ vaccine, and approval of this project. Exclusion criteria were apparent loss of or mistake in the questionnaire sheet.

### 2.4. Data Management Procedures

The data were entered into a validated database. The data-management group was responsible for data processing, in accordance with procedural documentation. Database locking occurred once quality assurance procedures were completed. After data were entered into the study database, a system of computerized data-validation checks was implemented and applied to the database on a regular basis. The study database was updated in accordance with the resolved queries.

The database was safeguarded against unauthorized access by established security procedures; appropriate backup copies of the database and related software files were maintained. Databases were backed up by the database administrator in conjunction with any updates or changes to the database. At critical junctures of the protocol (e.g., production of interim reports and final reports), data for analysis was locked and cleaned per established procedures.

### 2.5. Statistical Methods

The comparison between male and female groups was performed by χ^2^ analysis for each individual grade 0–4. To compare between gender and between allergic diseases, one-way ANOVA and intergroup comparison were conducted with the non-parametric method. We employed the Kruskal–Wallis test, followed by Scheffe’s single-step multiple-comparison procedure method. Values of *p* < 0.05 were considered statistically significant.

## 3. Results

### 3.1. Backgrounds of Vaccine Recipients

The characteristics of the participants in this study are listed in Table 1. The female participants outnumbered the male participants at a ratio of 3.62, because of the participation of high numbers of nurses and paramedical workers in the questionnaire survey. The age groups were well-balanced among those in their 20s, 30s, 40s, 50s, and >60s. The present or past histories of allergic diseases included BA (8.2%), AD (13.8%), FA (5.2%), pollinosis (44.8%), and hand eczema (6.3%). Hand eczema was included because it may represent a mild form of AD. These diseases overlapped in the individuals, such as those with BA plus AD, 2.3%; AD plus FA, 1.5%; AD plus pollinosis, 7.8%; and AD plus hand eczema, 3.2%. No allergic diseases were noted in 41.5% of the recipients.

### 3.2. Higher Grades of Adverse Reactions in the Second than in the First Dose and in Female than in Male Recipients

The grades of adverse reactions in each group were compared and analyzed statistically. In a comparison of the systemic reactions between the first and second doses, including headache, fatigability, muscle pain, joint pain, and fever, all were more severe in both male and female individuals receiving the second dose than in those given the first dose (Table 2), as reported previously [3]. Meanwhile, the local injection reactions of the two doses were comparable. When comparing between the male and female subjects, the intensities of the injection reactions (erythema and pruritus) for both the first and second doses were significantly higher (or tended to be higher) in the female subjects than in the male subjects. Notably, the systemic reactions were also more severe in the female recipients, as the grades of headache, fatigability, muscle pain, and joint pain for both the first and second doses were all higher in the female recipients than in the male recipients, consistent with the previous observations [7].

### 3.3. Higher Grades of Adverse Reactions in Recipients in Their 20s, 30s, and 40s than in Those in Their 50s or >60s 

**Figure 1:**
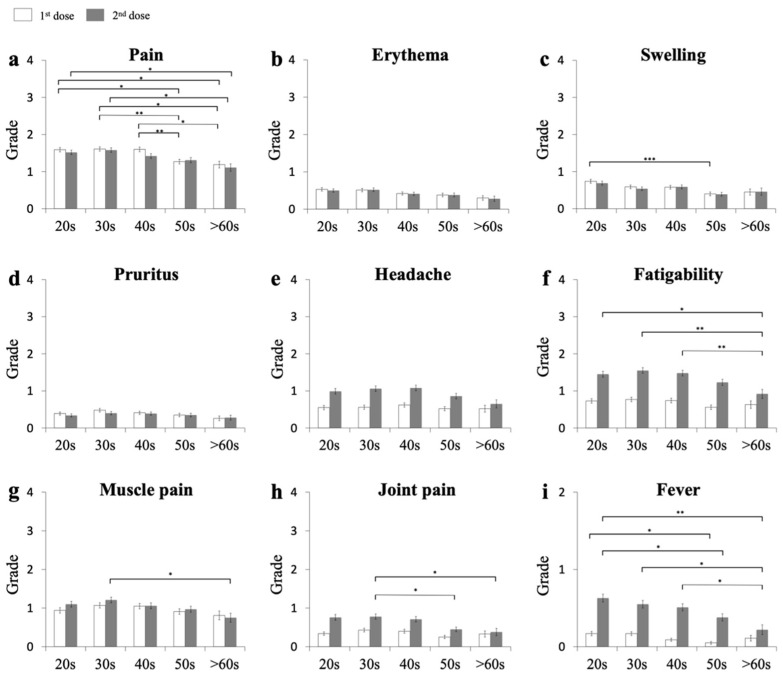
Local and systemic adverse reactions upon receiving first and second dose of vaccine in individuals in each 10-year age group. Local (**a**–**d**) and systemic (**e**–**i**) reactions are shown. Open bars represent the mean ± SE after the first dose and closed bars represent the mean ± SE after the second dose. * *p* < 0.05, ** *p* < 0.01, *** *p* < 0.001.

To test the age dependency of the adverse effects, the grades of the individual adverse reactions were examined for each 10-year age group (Figure 1). Following the Kruskal–Wallis test (*p* = 0.0001), the data were analyzed with Scheffe’s single-step multiple-comparison procedure method. The statistically significant differences are listed in Supplementary Table S2. For the first dose of the vaccination, there were no significant differences in the intensities of all the local and systemic reactions between those in their 50s and those >60. However, the severity grades of pain (Figure 1a), swelling (Figure 1c), and fever (Figure 1i) were significantly higher in the 20s, 30s, and 40s age groups than in the 50s and 60s age groups. The values were virtually comparable between 20s, 30s, and 40s age groups. For the second dose (Figure 1 and Supplementary Table S2), the grades of pain (Figure 1a), fatigability (Figure 1f), muscle pain (Figure 1g), joint pain (Figure 1h), and fever (Figure 1i) were significantly higher in the 20s, 30s and 40s age groups than in the 50s and 60s age groups. Thus, the younger recipients had higher severities of adverse reactions.

### 3.4. First-Dose Recipients with BA, but Not AD, FA, Pollinosis, or Hand Eczema, Show a Higher Grade of Fatigability

**Figure 2:**
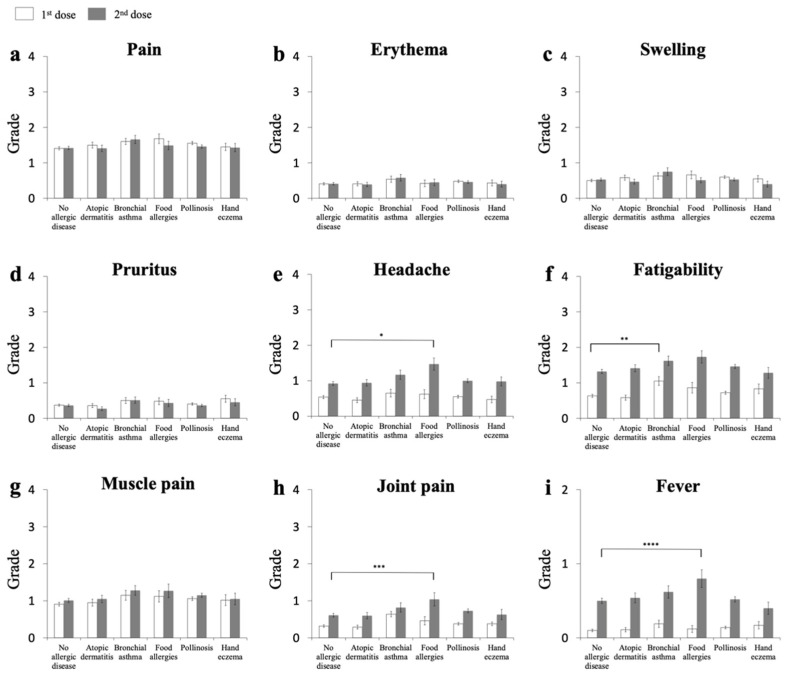
Local and systemic adverse reactions upon receiving first and second dose of vaccine in individuals with each of five allergic diseases and with no allergic disease. Local (**a**–**d**) and systemic (**e**–**i**) reactions are shown. Open bars represent the mean ± SE after the first dose and closed bars represent the mean ± SE after the second dose. * *p* = 0.0887, ** *p* = 0.0626, *** *p* = 0.338, **** *p* = 0.3027.

We compared the adverse reactions among the vaccine recipients with each of five allergic diseases and those without any of the diseases (Figure 2 and Supplementary Table S3). Following the Kruskal–Wallis test (*p* = 0.0880), the data were analyzed with Scheffe’s single-step multiple-comparison procedure method. Among the recipients of the first dose, there were no differences in the local injection reactions, including pain, erythema, swelling, and pruritus (Figure 2a–d), between the groups with AD, BA, FA, pollinosis, hand eczema, and no allergic disease. For the systemic symptoms, the degree of fatigability (Figure 2f, *p* = 0.0626) tended to be higher in subjects with BA than in those with no allergic diseases. There was no significant difference in headache, muscle pain, or fever among the six groups.

### 3.5. Second Dose Recipients with FA, but Not AD, BA, Pollinosis, or Hand Eczema, Show Higher Grades of Joint Pain and Fever

Following the Kruskal–Wallis test (*p* = 0.0841), the data were analyzed with Scheffe’s single-step multiple-comparison procedure method. Among the recipients of the second dose (Figure 2), again, there were no differences in the local injection reactions between the six groups. Regarding the general symptoms, there was a slight, but not negligible tendency for the degrees of headache (Supplementary Table S4), joint pain, and fever (data not shown) to be higher in subjects with FA than in the other groups (Figure 2h,i). 

### 3.6. Recipients with AD Show No Higher Adverse Reactions

Whether the skin lesions of recipients with AD are affected by the vaccine injection is uncertain. The severity of the local reactions was not high in those with AD compared to those with no allergic disease for the first or second dose of the vaccination (Figure 2). Rather, when we carefully observed the data for the second dose, the individuals with AD had lower mean values for all the local injection reactions (pain, erythema, swelling, and pruritus) than those with no allergic disease (Figure 2a–d). In addition, after the first dose, the recipients with AD tended to have lower severities of fatigability (Figure 2f) and joint pain (Figure 2h) than recipients with BA, suggesting that those with AD can tolerate the vaccination well.

## 4. Discussion

Through this questionnaire survey on the adverse reactions to receiving the first and second doses of the Pfizer–BioNTech vaccine, we investigated the effects of present or past histories of allergic diseases on the local and systemic reactions of the recipients. The people eligible for this study were workers in our hospital. The female-to-male ratio was 3.62, and the age groups were evenly distributed between those in their 20s, 30s, 40s, 50s, and >60s. Among the allergic diseases, we focused on AD, BA, FA, and pollinosis. Additionally, we listed hand eczema, which might potentially reflect mild AD. We could not correctly evaluate the incidence of anaphylaxis in this questionnaire, because most anaphylaxis-like symptoms are considered to represent vagal reflex, and discrimination between true anaphylaxis and other similar conditions is difficult within the limitations of a questionnaire. 

As was already known [3], all the systemic adverse reactions were highly graded for the second dose of the vaccination compared with the first dose, while the local injection reactions in the two doses were comparable. Given that the systemic adverse responses are mediated via the immunological mechanisms of sensitization and elicitation, the third dose might be even more severe. Future investigations may provide some insights. For both the first and second doses, the local and systemic reactions were more severe in female than in male recipients, and the younger age groups exhibited worse reactions, consistent with the previous reports [7]. 

While the existence of present or past histories of allergic diseases did not exacerbate the local injection reactions, some of the systemic reactions were intensified when the subjects had BA, FA, pollinosis, or hand eczema, but not AD. Rather, AD reduced the severity of the reactions, as assessed by the mean intensities. In BA patients, the frequencies of severity grades above “mild” were 35.6–68.9% for each systemic adverse reaction after the second dose, and those for grades above “high” were 9.1–21.4%, indicating that the adverse effects were mostly mild, as is consistent with previous observations [8]. However, the severity of fatigability was significantly higher in our recipients with BA after the first dose than in the non-allergic recipients. The reason for the BA-associated increase in the intensity of some of the adverse reactions remains unclear from this study. 

Virus-induced T-cell-mediated heterologous immunity has been widely observed in a variety of settings, and it can either protect from or lead to immunopathology against other antigens [9]. The occurrence of a considerable number of skin lesions upon SARS-CoV2 vaccination mimics the virus infection, as these lesions are also seen in COVID-19. BA has not been shown to be a risk factor for COVID-19 in several cohort studies [10]. Rather, according to the UK Biobank, patients with allergic BA had a lower risk of severe COVID-19, as compared to non-allergic patients [11]. It has been hypothesized that SARS-CoV-2 has a degree of homology with the protein sequence of allergens, which may lead to the generation of cross-reactive T-cell epitopes [12]. Pre-existing T cells specific to such cross-reactive, allergen-derived epitopes possibly modulate the COVID-19 outcome via T-cell responses to the virus peptides. By an in silico analysis, Balz et al. identified allergens potentially cross-reactive with SARS-CoV-2 T-cell epitopes, suggesting that patients with BA may be affected by a heterologous immune response against SARS-CoV-2 [12]. They highlighted the epitopes from the *Dermatophagoides* species *Aspergillus fumigatus* and *Phleum pratense*. In our study, we found significantly more severe systemic reactions induced by the first, but not the second, dose of vaccination. Given the sequence homology between SARS-CoV-2 and clinically relevant respiratory allergens, our observation might support the idea that BA patients are already sensitized with certain respiratory antigens, thereby inducing higher severities of systemic adverse reactions. In this context, it is an interesting observation that SARS-CoV-2-reactive CD4^+^ T cells were present in 40–60% of the virus-unexposed individuals [13]. Nevertheless, it remains uncertain whether such epitopes shared by the allergens and the virus sequence can promote the adverse reactions, or rather, soften the symptoms in the vaccine recipients. 

On the other hand, the effects of allergic diseases on the severity of COVID-19 have been studied, focusing on the expression of the virus receptor angiotensin-converting enzyme 2 (ACE2) [14]. It is possible that the SARS-CoV2 vaccination yields the expression of the viral spike protein, which can bind to ACE2. A study using adult bronchial brush samples showed an inverse correlation between ACE2 gene expression and a Th2 dependent gene expression signature [15]. Similarly, nasal epithelial cells from children with atopic asthma express significantly lower levels of ACE2 [16]. These findings raise the possibility that the type 2 immunological state protects from the virus infection via downregulating its receptor. 

In FA patients, joint pain and fever tended to be more severe than in the non-allergic group after the second dose. The mechanism underlying this association remains unclear, but it seems unlikely that the food components and the SARS-CoV-2 sequence cross-reacted with each other. Since the sequence homology between SARS-CoV-2 and food allergens is poor, food allergens appear to be of lower importance than respiratory allergens in the cross-reactivity [12]. 

When patients with AD suffer from COVID-19, they may exhibit less severe symptoms [17]. Interestingly, our present study showed that the vaccine recipients with AD tended to have rather lower intensities of local adverse reactions. Thus, AD may provide a protective immunological state in the expression of skin reactions toward SARS-CoV-2 infection and its vaccine. Given that AD confers a type 2 inflammation [18], interleukin (IL)-4 and IL-13 possibly depress vaccine-associated type 1 inflammation. Alternatively, epithelial cells in AD patients express lower levels of ACE2 [15,16]. We observed significantly less-severe systemic symptoms in AD than in BA patients, although both AD and BA are type 2-skewing diseases [19]. There is evidence for a reciprocal relationship between atopic diseases and the production of type I and III interferons in response to viral infections [14]. Provided that atopic conditions are not a significant risk factor for severe clinical courses of COVID-19, the epitope homology, if any, most likely plays a protective role [20]. In this respect, the homology between SARS-CoV-2 T-cell epitopes and *Dermatophagoides* species epitopes should be noted, because of the clinically relevant and immunoregulatory therapeutic role of *Dermatophagoides pteronyssinus* and *farinae* in AD patients [21].

Because of the limitations of the retrospective questionnaire survey, our findings are not sufficiently conclusive. However, this study may provide some information on the epidemiology of vaccine allergy and serve as a basis for expert opinion in conjunction with public health guidance. In particular, the results contribute to guidelines for the care of individuals with different allergy histories, so that they can safely receive their vaccine. Practically, the streamlining of the vaccination process to improve the operation at the site of vaccination is essential. In Japan, there are two opportunities for vaccine recipients to ask doctors if individuals are allowed to be vaccinated even with complications. One is that, in advance of vaccination, recipients can obtain permission from their family doctors or vaccination physicians. The other opportunity comes at the vaccination site, where doctors consult the patients’ medical interview sheet. In any case, there is a high incidence of questions regarding whether allergic diseases influence the adverse effects of a given vaccine. While there are guidelines and recommendations for anaphylaxis sufferers [5], no recommendations or evidence for the effects of allergic diseases have been provided. The results of our study may be helpful to respond to the recipients’ questions.

Among approximately 1000 subjects, nearly 60% of whom had allergic diseases, no definite adverse effects of allergic diseases on the SARS-CoV2 vaccination were found. It is notable that individuals with pre-existing allergic diseases are not prone to having allergic side effects to the vaccination.

## Figures and Tables

**Table 1:** Characteristics of participants in this study.

Variable	Recipients
N of recipients	955
Sex	
Male, n (%)	203 (21.3)
Female, n (%)	734 (76.9)
Unknown, n (%)	18 (1.9)
Age	
20s, n (%)	235 (24.6)
30s, n (%)	230 (24.1)
40s, n (%)	230 (24.1)
50s, n (%)	186 (19.5)
>60, n (%)	74 (7.7)
No allergic disease, n (%)	396 (41.5)
History of AD ^1^, n (%)	132 (13.8)
History of BA ^2^, n (%)	78 (8.2)
History of FA ^3^, n (%)	50 (5.2)
History of pollinosis, n (%)	428 (44.8)
History of hand eczema, n (%)	60 (6.3)

^1^ AD: atopic dermatitis; ^2^ BA: bronchial asthma; ^3^ FA: food allergies.

**Table 2:** Comparison of grades of each adverse reaction between male and female recipients.

Adverse Reactions	Male (n = 203 [21.3%])					Female (n = 734 [76.9%])					*p* Value
First dose	Grade *					Grade *					
Local injection reactions	0	1	2	3	4	0	1	2	3	4	
Pain, n (%)	27 (13.3)	89 (43.8)	62 (30.5)	23 (11.3)	2 (1.0)	92 (12.5)	284 (38.7)	242 (33.0)	114 (15.5)	2 (0.2)	0.26
Erythema, n (%)	148 (72.9)	40 (19.7)	13 (6.4)	2 (1.0)	0	455 (62.0)	213 (29.0)	61 (8.3)	5 (0.7)	0	0.0295
Swelling, n (%)	131 (64.5)	43 (21.2)	13 (6.4)	2 (1.0)	0	414 (56.4)	213 (29.0)	99 (13.5)	8 (1.1)	0	0.13
Pruritus, n (%)	169 (83.3)	23 (11.3)	11 (5.4)	0	0	487 (66.3)	167 (22.8)	80 (10.9)	0	0	<0.0001
Systemic reactions	0	1	2	3	4	0	1	2	3	4	
Headache, n (%)	164 (80.8)	23 (11.3)	16 (7.9)	0	0	432 (58.9)	166 (22.6)	102 (13.9)	32 (4.4)	2 (0.2)	<0.0001
Fatigability, n (%)	140 (69.0)	39 (19.2)	23 (11.3)	1 (0.5)	0	382 (52.0)	184 (25.1)	126 (17.2)	41 (5.6)	1 (0.1)	0.0001
Muscle pain, n (%)	116 (57.1)	48 (23.6)	26 (12.8)	11 (5.4)	2 (1.0)	298 (40.6)	176 (24.0)	180 (24.5)	75 (10.2)	5 (0.7)	0.0001
Joint pain, n (%)	173 (85.2)	21 (10.3)	7 (3.4)	1 (0.5)	1 (0.5)	527 (71.8)	147 (20.0)	37 (5.0)	22 (3.0)	1 (0.1)	0.0012
Fever, n (%)	184 (90.6)	17 (8.4)	2 (1.0)			684 (93.2)	82 (11.2)	4 (0.5)			0.415
Anaphylaxis (or vagal reflex), n (%)	201 (99.0)	2 (1.0)				724 (98.6)	10 (1.4)				0.94
Second dose	Grade *					Grade *					
Local injection reactions	0	1	2	3	4	0	1	2	3	4	
Pain, n (%)	36 (17.7)	81 (40.0)	64 (31.5)	21 (10.3)	1 (0.5)	116 (15.8)	287 (39.1)	209 (28.5)	119 (16.2)	0	0.086
Erythema, n (%)	150 (73.9)	37 (18.2)	12 (5.9)	4 (2.0)	0	472 (64.3)	191 (26.0)	58 (8.0)	10 (1.4)	0	0.061
Swelling, n (%)	136 (67.0)	41 (20.2)	21 (10.3)	5 (2.5)	0	435 (59.3)	191 (26.0)	88 (12.0)	17 (2.3)	0	0.24
Pruritus, n (%)	172 (84.7)	23 (11.3)	8 (3.9)	0	0	507 (69.1)	153 (20.8)	64 (8.7)	7 (1.0)	0	0.0002
Systemic reactions	0	1	2	3	4	0	1	2	3	4	
Headache, n (%)	144 (70.9)	29 (14.3)	20 (9.9)	9 (4.4)	1 (0.5)	306 (41.7)	144 (19.6)	182 (24.8)	94 (12.8)	5 (0.7)	<0.0001
Fatigability, n (%)	93 (45.8)	43 (21.2)	36 (17.7)	27 (13.3)	3 (1.5)	194 (26.4)	164 (22.3)	201 (27.4)	159 (21.7)	13 (1.8)	<0.0001
Muscle pain, n (%)	104 (51.2)	42 (20.7)	39 (19.2)	16 (7.9)	2 (1.0)	296 (40.3)	160 (21.8)	161 (21.9)	108 (14.7)	6 (0.8)	0.03
Joint pain, n (%)	160 (78.8)	21 (10.3)	12 (5.9)	10 (4.9)	0	440 (60.0)	123 (16.8)	93 (12.7)	69 (9.4)	6 (0.8)	0.0001
Fever, n (%)	137 (67.5)	45 (22.2)	21 (10.3)			452 (61.6)	180 (24.5)	97 (13.2)			0.42
Anaphylaxis (or vagal reflex), n (%)	202 (99.5)	1 (0.5)				720 (98.1)	11 (1.5)				0.43

* The numbers of subjects with individual adverse reactions are listed for each grade (0–4). Parentheses indicate the frequencies (%).

## Data Availability

The data in this study are available if one asks the corresponding author (H.M.) to approach them.

## Supplementary Materials

The following are available online at https://www.mdpi.com/article/10.3390/vaccines9121421/s1, Table S1: questionnaire on the adverse effects of SARS-CoV-2 vaccination, Table S2: Multiple comparison of intensities in each adverse reaction between individual age groups. Table S3: Multiple comparison of intensities in fatigability after the first dose among the recipients with each allergic disease and those without any of the diseases. Table S4: Multiple comparison of intensities in headache after the second dose among the recipients with each allergic diseases and those without any of the diseases.
