# Supplementary material for: Questionnaire Survey of Possible Association of Allergic Diseases with Adverse Reactions to SARS-CoV-2 Vaccination
Source: Vaccines (Basel). 2021 Dec 1;9(12):1421. doi: 10.3390/vaccines9121421 (PMC8708946; doi:10.3390/vaccines9121421)
Supplement: Supplementary file 1 [file vaccines-09-01421-s001.zip › Suppl Table S1.pdf]

Suppl Table S1.

Questionnaire to Adverse Effects of SARS-CoV-2 Vaccination

Please put check mark ☑ on each question

※Refer to another sheet to respond to Question 4 and 5

1. Your gender and age

【Gender】 □ Male □Female □Other

【Age (years)】 □ -19 □ 20-29 □ 30-39 □ 40-49 □ 50-50 □ 60-69 □ 70-79 □ 80-

2. Present illness or past histories of diseases

□ Atopic dermatitis □ Bronchial asthma □ Food allergy □ Pollinosis □ Hand eczema

Other allergic diseases（ ）

3. If you took medicine related to Question 2 diseases upon vaccination, please specify it.

Name(s) of medicine（ ）

4. **Adverse effects after 1st dose**	Mild	Moderate	High	Severe
□ Pain at injection site	□	□	□	□
□ Redness at injection site	□	□	□	□
□ Swelling at injection sit	□	□	□	□
□ Itching at injection site	□	□	□	□
□ Headache	□	□	□	□
□ Tiredness	□	□	□	□
□ Muscle pain	□	□	□	□
□ Joint pain	□	□	□	□
□ Fever	□ 37.5℃-37.9℃		□ 38.0℃-	
□ Anaphylaxis				

Other symptoms（ ）

5. **Adverse effects after 2nd dose**	Mild	Moderate	High	Severe
□ Pain at injection site	□	□	□	□
□ Redness at injection site	□	□	□	□
□ Swelling at injection sit	□	□	□	□
□ Itching at injection site	□	□	□	□
□ Headache	□	□	□	□
□ Tiredness	□	□	□	□
□ Muscle pain	□	□	□	□
□ Joint pain	□	□	□	□
□ Fever	□ 37.5℃-37.9℃		□ 38.0℃-	
□ Anaphylaxis				

Other symptoms（ ）

6. If you took medicine before vaccination, please specify it.

【Before 1st dose】 Medicine（ ）

【Before 2nd dose】 Medicine（ ）

７. If you took medicine after vaccination for Question 4 and 5 symptoms, please specify it.

【After 1st dose】 Medicine（　　　　　　　　　　　　）

【After 2nd dose】 Medicine（　　　　　　　　　　　　）

８. If you have any opinions or views, please tell us.

