# Supplementary material for: Questionnaire Survey of Possible Association of Allergic Diseases with Adverse Reactions to SARS-CoV-2 Vaccination
Source: Vaccines (Basel). 2021 Dec 1;9(12):1421. doi: 10.3390/vaccines9121421 (PMC8708946; doi:10.3390/vaccines9121421)
Supplement: Supplementary file 1 [file vaccines-09-01421-s001.zip › Suppl Table S2.pdf]

Suppl Table S2. Multiple comparison of intensities in each adverse reaction between individual age groups

Adverse effects	Age group 1	Age group 2	χ^2 Value	*P* Value
First dose				
Local Injection reactions				
Pain	20s	50s	12.6521	0.0131
	20s	>60s	11.0491	0.0260
	30s	50s	13.8466	0.0078
	30s	>60s	11.8974	0.0181
	40s	50s	13.3937	0.0095
	40s	>60s	11.5829	0.0207
Swelling	20s	50s	22.4743	0.0002
Systemic reactions				
Fever	20s	50s	10.5470	0.0322
Second dose				
Local Injection reactions				
Pain	20s	>60s	9.8609	0.0428
	30s	>60s	13.1379	0.0106
Systemic reactions				
Fatigability	20s	>60s	11.8102	0.0188
	30s	>60s	16.8911	0.0020
	40s	>60s	13.5932	0.0087
Muscle pain	30s	>60s	9.6616	0.0465
Joint pain	30s	50s	11.4283	0.0221
	30s	>60s	9.6268	0.0472
Fever	20s	50s	11.4900	0.0216
	20s	>60s	18.3107	0.0011
	30s	>60s	12.3980	0.0146
	40s	>60s	10.8564	0.0282
