# Supplementary material for: Questionnaire Survey of Possible Association of Allergic Diseases with Adverse Reactions to SARS-CoV-2 Vaccination
Source: Vaccines (Basel). 2021 Dec 1;9(12):1421. doi: 10.3390/vaccines9121421 (PMC8708946; doi:10.3390/vaccines9121421)
Supplement: Supplementary file 1 [file vaccines-09-01421-s001.zip › Suppl Table S3.pdf]

Suppl Table S3. Multiple comparison of intensities in fatigability after the first dose among the recipients with each allergic diseases and those without any of the diseases

Disease group 1	Disease group 2	χ^2 Value	*P* Value
No allergic disease	AD	0.3222	0.9972
No allergic disease	BA	10.4835	0.0626
No allergic disease	FA	1.6846	0.8908
No allergic disease	Pollinosis	1.9760	0.8525
No allergic disease	Hand eczema	1.5666	0.9053
AD	BA	10.2690	0.0680
AD	FA	2.2982	0.8065
AD	Pollinosis	2.4166	0.7890
AD	Hand eczema	2.1884	0.8225
BA	FA	1.2967	0.9353
BA	Pollinosis	6.0605	0.3004
BA	Hand eczema	1.7583	0.8815
FA	Pollinosis	0.4194	0.9948
FA	Hand eczema	0.0125	1.0000
Pollinosis	Hand eczema	0.2990	0.9977

Abbreviations. AD: atopic dermatitis; BA: bronchial asthma; FA: food allergies.
