# Supplementary material for: Questionnaire Survey of Possible Association of Allergic Diseases with Adverse Reactions to SARS-CoV-2 Vaccination
Source: Vaccines (Basel). 2021 Dec 1;9(12):1421. doi: 10.3390/vaccines9121421 (PMC8708946; doi:10.3390/vaccines9121421)
Supplement: Supplementary file 1 [file vaccines-09-01421-s001.zip › Suppl Table S4.pdf]

Suppl Table S4. Multiple comparison of intensities in headache after the second dose among the recipients with each allergic diseases and those without any of the diseases

Disease group 1	Disease group 2	χ^2 Value	*P* Value
No allergic disease	AD	0.0891	0.9999
No allergic disease	BA	3.2191	0.6663
No allergic disease	FA	9.5591	0.0887
No allergic disease	Pollinosis	1.2529	0.9397
No allergic disease	Hand eczema	0.4915	0.9924
AD	BA	1.8152	0.8741
AD	FA	6.8298	0.2336
AD	Pollinosis	0.2289	0.9988
AD	Hand eczema	0.1840	0.9993
BA	FA	1.7671	0.8803
BA	Pollinosis	1.3878	0.9256
BA	Hand eczema	0.5459	0.9903
FA	Pollinosis	6.6935	0.2445
FA	Hand eczema	3.7153	0.5911
Pollinosis	Hand eczema	0.0191	1.0000

Abbreviations. AD: atopic dermatitis; BA: bronchial asthma; FA: food allergies.
